# Exercise Promotion in Saudi Arabia: Understanding Personal, Environmental, and Social Determinants of Physical Activity Participation and Well-Being

**DOI:** 10.3390/ijerph20043554

**Published:** 2023-02-17

**Authors:** Naif Albujulaya, Clare Stevinson

**Affiliations:** 1 School of Sport, Exercise and Health Sciences, Loughborough University, Loughborough LE11 3TU, UK; 2 Department of Physical Education, College of Education, King Faisal University, Al-Ahsa 31982, Saudi Arabia

**Keywords:** physical activity, Saudi Arabia, barriers, nature relatedness, well-being, health, exercise

## Abstract

Physical activity promotion has received increasing attention globally due to the considerable benefits of regular activity for population health and well-being. In Saudi Arabia, government strategy explicitly aims to increase physical activity participation among residents. This study assessed the barriers to physical activity among the general Saudi population including any age and gender differences and examined the contribution of contextual factors and nature relatedness to health and well-being. A representative sample of 1046 Saudi adults (aged 18 years and above) completed an online survey that included four validated scales: the International Physical Activity Questionnaire—short form, the Exercise Benefits/Barriers Scale, the World Health Organization Five Well-Being Index and the Nature Relatedness Scale. Analyses indicated that young Saudi adults perceived more barriers than middle-aged and older adults, but few gender differences were observed. Furthermore, exercising outdoors, with other people and via sport predicted higher levels of mental well-being, as did nature relatedness. Therefore, developing a comprehensive strategy package that includes the development of outdoor environments for all age groups across regions and fostering a connection with nature may be particularly effective to improve the health and well-being of Saudi adults.

## 1. Introduction

There is indisputable evidence that physical activity plays an important role in the prevention of many diseases, including heart disease, obesity, some cancers, and diabetes [1] and is associated with lower mortality rates [2]. In addition, physical activity is recognised as vital in the maintenance of mental health and enhancing well-being [3].

Globally, 27.5% of the adult population is estimated to be insufficiently active [4], with this phenomenon being more marked among females (31.7%) and in high-income countries (36.8%). The World Health Organization (WHO) encourages all countries to develop policies to promote physical activity [5] in an attempt to reverse current trends in ill health and its associated costs.

In Saudi Arabia, several diseases related to physical inactivity are prevalent and exceed the respective global rates, such as heart disease [6] and diabetes [7]. Similarly, rates of obesity and overweight are notably high [8], and surveillance research has indicated that 60.1% of men and 72.9% of women have low levels of physical activity [9]. These figures highlight the need to understand the factors influencing physical activity behaviour among Saudi adults to inform health promotion strategies.

Research in neighbouring countries, such as Oman, Qatar, Kuwait and the United Arab Emirates, has highlighted that there is insufficient research in the Gulf region on understanding the barriers to physical activity [10,11,12,13]. In particular, low levels of activity among young people and females have been highlighted as priorities to study [10]. In Saudi Arabia specifically, there is a current lack of research on the barriers to physical activity among the general population. For college students, lack of time and facilities have been consistently identified as the most common obstacles [14,15,16,17,18,19,20]. Research with patients attending primary care clinics has suggested that low energy and insufficient financial resources may hinder physical activity [21,22]. In addition to these data on specific sectors of the population, it is necessary to gain a broader understanding of physical activity barriers across all population groups. 

In particular, due to the rapid changes in Saudi Arabia in the last five years, additional research is warranted to further broaden the understanding of opportunities and difficulties associated with physical activity among Saudi females. The Saudi government has shown strong support for women’s sports, especially after Vision 2030 was launched [23]. This can be seen in many of the decisions made by the government in the last few years, for example, allowing sports in girls’ schools [24], licensing women’s gyms and establishing women’s sports competitions [25]. However, limited attention has been given to examining the impact of the recent changes on physical activity participation among adult Saudi women.

One aspect of physical activity promotion that has gained increasing attention in recent years is the value of the outdoor environment [26]. Using existing public spaces as locations for exercise helps address some of the barriers relating to access to facilities and cost. Additionally, several benefits to physical and mental well-being have been demonstrated through exercising outdoors in natural settings in comparison to exercising indoors or in urban locations. These include increased vitamin D intake [27,28], lowered blood pressure [29] and cortisol [30], and improved cognition and mood [31]. Physical activity in natural environments is also associated with greater enjoyment and adherence [32]. This growing evidence supporting outdoor physical activity as accessible, enjoyable and beneficial is promising in terms of health promotion planning. However, to date, most such research has been conducted in Europe, Japan and the United States. The role of the environment in physical activity participation in Saudi Arabia has not been investigated.

An interesting consideration with respect to the relationship between outdoor exercise and well-being and adherence benefits is the level of connection to nature held by individuals. Nature connectedness refers to a stable characteristic reflecting interest in nature and desire for contact with the natural world. Higher levels of this characteristic are associated with greater well-being and vitality [33], as well as life satisfaction and self-esteem [34]. It is possible that the potential value of outdoor natural environments for promoting physical activity is dependent on the degree of nature connectedness held by residents.

This study was aimed at addressing some of the understudied aspects of physical activity promotion in Saudi Arabia. The primary objective was to assess the strength of barriers to physical activity among the general Saudi population and identify any differences based on gender and age. The secondary objective was to examine the contribution of physical activity context and nature connectedness to health and well-being.

## 2. Methods

### 2.1. Participants

A cross-sectional, population-based online survey was used for this study, collecting data on well-being, general health, nature relatedness and physical activity variables. A random sample of 1046 Saudi adults (18 years and above) was recruited in March 2021 by an international research agency from a national panel of residents weighted by key demographic factors (age, gender, nationality and city) to ensure urban representativeness. Participants needed to be a Saudi citizen, 18 years of age or older, able to read and have access to the Internet to be included in this study. Ethical clearance was secured from Loughborough University’s Ethics Approvals (Human Participants) Sub-Committee (SSEHS-1708), and all participants provided informed consent before taking part.

### 2.2. Measures

Physical activity levels were assessed with the Arabic version of the short form of the International Physical Activity Questionnaire (IPAQ-SF) [35]. Respondents are asked to report the duration and frequency of vigorous and moderate-intensity physical activity and walking over the previous seven days. The IPAQ-SF is widely used in physical activity surveillance research due to its low participant burden, but it is recognised that it leads to higher estimates of physical activity than recorded by objective devices [36].

Perceived barriers to physical activity were assessed with the Exercise Benefits/Barriers Scale (EBBS) [37]. The EBBS includes 29 items on the benefits of physical activity and 14 items on the barriers to physical activity. Only the latter 14 items on barriers were used in the current study. Sechrist et al. [38] grouped the barrier items into four categories: exercise milieu (6 items relating to location, cost, and embarrassment), time expenditure (3 items), physical exertion (3 items) and family discouragement (2 items). The EBBS is rated on a 4-point Likert scale ranging from 1 (strongly agree) to 4 strongly disagree. High internal consistency (Cronbach alpha 0.89) has been demonstrated for the barriers scale [38]. Since no Arabic version of the EBBS scale was available, we translated the scale from English to Arabic. To ensure the content validity of the Arabic version, we followed the required steps outlined by Bryman [39]. The Arabic version was sent to four sport science experts who were fluent in Arabic and English, accompanied by the objectives of the study and the purpose of the questionnaire, to gather their assessments on the accuracy of the translated scale. The experts’ comments on the translated version were incorporated to improve the wording of the scale. The revised version was piloted with a sample of Saudi adults across multiple regions (n = 62). A Cronbach alpha of 0.80 indicated acceptable internal consistency, and this was confirmed in the subsequent study sample (Cronbach alpha of 0.91). The EBBS does not include weather conditions as a barrier to physical activity. However, since the weather has been shown to influence exercise behaviours [40], particularly in hot countries [41], an item relating to weather conditions as a potential barrier was added for the current study.

Physical activity context variables were assessed by the inclusion of six statements rated on the same 4-point scale as the EBBS. These related to the extent that physical activity that was performed: (1) outdoors; (2) indoors; (3) alone; (4) with other people; (5) for sport/recreation; (6) as part of lifestyle.

Psychological well-being was assessed via the Arabic version of the World Health Organization Five Well-Being Index (WHO-5) [42]. The index consists of five statements rated on a 6-point Likert scale, ranging from 0 (at no time) to 5 (all of the time). Examples of items include: ‘I have felt cheerful and in good spirits’ and ‘I have felt active and vigorous’. Scores are summed, with a maximum score of 25 representing the best possible well-being. The WHO-5 has strong psychometric qualities including good construct validity [43]. High internal consistency was demonstrated for the current sample (Cronbach alpha of 0.89). A standard question on perceived general health was included based on the World Health Survey Saudi Arabia [44], in which participants reported their general health as excellent, very good, good, fair or poor.

Nature relatedness was assessed with the short-form version of the Nature Relatedness Scale (NR-6) to estimate the participants’ relationship to nature [45]. The NR-6 scale consists of 6 items rated on a 5-point Likert scale ranging from 1 (disagree strongly) to 5 (agree strongly). Example items include ‘my relationship to nature is an important part of who I am’ and ‘I take notice of wildlife wherever I am’. The NR-6 compares favourably with the original full-length scale for convergent validity and has strong internal consistency and test–retest reliability [45]. Because no Arabic version of the NR-6 scale was available, we followed the same steps as with the EBBS to develop an Arabic version to ensure it was reliable and valid. An acceptable level of internal consistency was demonstrated for the pilot sample (Cronbach alpha of 0.74) and for the study sample (Cronbach alpha of 0.82).

### 2.3. Statistical Analysis

Data cleaning and analysis were performed using SPSS version 28.0 (IBM SPSS Statistics, Chicago, FL, USA). The IPAQ scoring guidelines [46] were followed for truncation and to remove outliers before calculating the physical activity levels of participants as metabolic equivalent of task (MET) minutes per week. MET values of 8.0 for vigorous physical activity, 4.0 for moderate-intensity activity and 3.3 for walking were applied to the average minutes per week (i.e., frequency multiplied by duration of sessions) of each intensity category and summed. Median values and interquartile ranges were calculated for physical activity data.

Descriptive statistics for other variables included means, standard deviations, and frequencies. Differences based on gender and age groups in physical activity barriers and context variables were examined with two-way analysis of variance (ANOVA). Associations between well-being, general health, nature relatedness and physical activity variables (level, barriers, and context) were examined with Pearson’s correlations. Finally, a multiple linear regression test was used to predict the value of well-being based on the value of the other correlated variables. The significance level was set at *p* < 0.05 for all analyses.

## 3. Results

Sociodemographic characteristics of the participants are shown in Table 1. Among the sample, 61% were male and 39% were female. The mean age was 37.0 ± 11.6. After following the IPAQ scoring guidelines, data from 28 participants were excluded, leaving a final sample of 1018 for analyses involving physical activity. The overall sample has a median PA level of 1377 (IQR = 2274) MET minutes/week.

**Figure 1:**
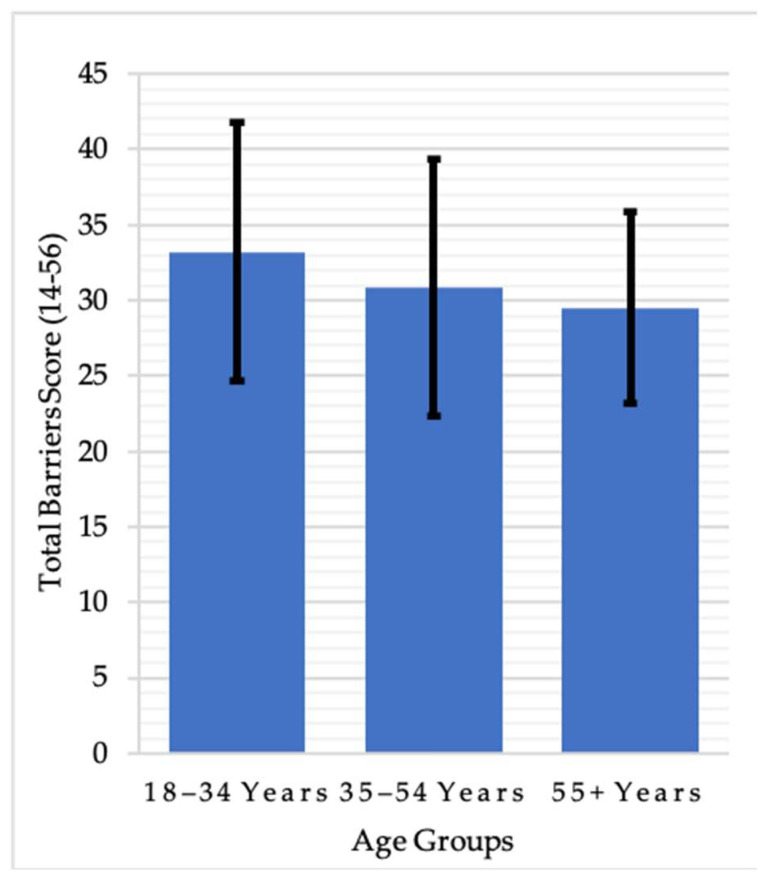
Differences in total barrier scores between age groups.

Table 2 presents the descriptive statistics for exercise barrier variables for the total sample and divided by gender and age group. Two-way ANOVAs were used to examine differences between groups. Despite higher values for most barriers among females, main effects analysis showed that only physical exertion barriers significantly differed between males and females (F(1,1044) = 4.608, *p* = 0.032). For age, the analysis revealed that there were significant differences for all types of barriers based on age group: exercise milieu (F(2,1040) = 7.655, *p* < 0.001), time expenditure (F(2,1040) = 4.506, *p* = 0.011), physical exertion (F(2,1040) = 8.993, *p* < 0.001), family discouragement (F(2,1040) = 5.511, *p* = 0.004), total barriers (F(2,1040) = 8.233, *p* < 0.001), and weather conditions (F(2,1040) = 4.506, *p* = 0.011). Comparison of the Bonferroni adjusted pairwise mean ranks highlighted that younger adults aged 18–34 years had significantly higher scores for all types of barrier than the older age groups, except for time expenditure and weather barriers, which only significantly differed from mid-life adults aged 35–54 years (Figure 1). A significant interaction effect for gender and age existed only for family discouragement barriers (F(2,1040) = 6.566, *p* = 0.001). Examining gender differences within age categories by Bonferroni post hoc test indicated that older females (55+ years) experienced more family discouragement then older males (*p* < 0.001, d = 0.37).

Table 3 displays the means for the physical activity context variables based on gender and age group. The main effects of gender were significant for all context variables. Outdoor exercise values were higher for men than women (F(1,870) = 39.269, *p* < 0.001), while indoor exercise was favoured by females (F(1,870) = 13.206, *p* < 0.001). Similarly, males exercised more with other people (F(1,870) = 32.354, *p* < 0.001), while females exercised alone (F(1,870) = 6.257, *p* = 0.013). Finally, the rate of exercising via playing sport was higher among males (F(1,870) = 13.304, *p* < 0.001), while lifestyle activity was preferred by females (F(1,870) = 4.076, *p* = 0.044). However, the only two significant differences by age were that younger adults (18–34 years) preferred exercising indoors (F(2,869) = 13.563, *p* < 0.001) and alone (F(2,869) = 9.640, *p* < 0.001) more than older groups. A two-way ANOVA indicated that there were statistically significant interactions between the effects of gender and age on exercising outdoors (F(2,866) = 3.868, *p* = 0.021) and exercising with others (F(2,866) = 6.390, *p* = 0.002). Bonferroni post hoc analysis revealed that within the 18–34 age group, males were significantly more likely to exercise outdoors (*p* < 0.001, d = 0.58) and with others (*p* < 0.001, d = 0.54) than females. Within the 35–54 age group, Bonferroni post hoc analysis showed that males preferred to exercise outdoors (*p* = 0.003, d = 0.33) and with others (*p* < 0.001, d = 0.39) significantly more than females.

Table 4 summarises the descriptive data for the well-being, nature relatedness and general health variables and the associations with physical activity level, barriers and context. Significant positive relationships were indicated between well-being and physical activity level, general health, nature relatedness and five physical activity context variables (outdoor activity, indoor activity, other people, lifestyle activity and sport-related activity). These variables were entered into the multiple linear regression analysis as possible predictors of well-being. It was found that these variables explain a significant amount of the variance in well-being scores (F (6,837) = 51.982, *p* < 0.001), with an R square of 0.27, suggesting that these eight predictors can explain 27% of the variance in participants’ well-being. The analysis showed that six variables were significant independent predictors of well-being: PA levels (β = 0.17), NR-6 score (β = 0.25), general health (β = 0.23), exercising with others (β = 0.13), exercising outdoors (β = 0.08) and exercising via playing certain sports (β = 0.09). These results are shown in Table 5.

## 4. Discussion

Despite the appreciable influence of physical activity on public health, insufficient attention has been paid to identifying the barriers that hinder the general Saudi population from participating in physical activity. In addition, no research has investigated the impact of physical activity context and nature connectedness on the health and well-being of the Saudi population. This study uncovered the barriers to physical activities experienced by Saudi adults and found that young adults experienced more barriers than mid-life and older adults, while few significant differences existed between men and women. Furthermore, correlation analysis demonstrated that physical activity contexts and nature connectedness have relationships with health and well-being.

The analysis of barriers to exercise indicated that Saudi females had higher scores for physical exertion than males. However, it highlighted that overall, the differences in perceived barriers between males and females are not significant in terms of social or environmental barriers but only in terms of individual barriers. These findings seem inconsistent with other recent research, which concluded that the non-availability of specialized facilities is a barrier that Saudi females might face more than Saudi males [47]. However, this may reflect the lack of female gyms available at the time of data collection for the previous study conducted in 2018, since the introduction of women’s gyms was only allowed by the government in the middle of 2017 [48]. Prior to this, there was a general discouragement of exercise among Saudi females due to strong cultural and religious expectations [22,47,49,50]. This is reflected in higher family discouragement barriers among older women than men, while no gender differences existed between younger and mid-life participants. Since Vison 2030 was launched, there have been rapid changes in Saudi Arabian society, especially among younger populations, who are the main target of government strategies. However, for older females, traditional values may persist, including beliefs that women should not leave the house except for necessity and that women are solely responsible for the care of children. This supports previous research that has pointed to the negative influence of family on older females’ participation in physical activity [51,52]. Collectively, these findings suggest that concentrating on enhancing personal motivation for physical activities among Saudi females must be a priority of the Saudi government, and extra attention should be given by the government to developing programmes and strategies to enhance a change in the culture of physical activity among older adults in particular.

The results of this study indicate that young participants (18–34 years) experienced more barriers than mid-life and older adults. In accordance with the present results, previous studies conducted among Malaysians [53], UK citizens [54], Australians [55] and Korean-Americans [56] have demonstrated that young adults are more likely to experience barriers to physical activities than older adults. There are several possible explanations for these results. It is known that individuals in this age group are going through transitional phases, such as starting college/university education, seeking jobs and helping parents with family responsibilities, which may lead to financial instability, time limitations and fatigue. Thus, having the financial capacity, free time and energy to engage in physical activities can be challenging compared with other groups. In addition, individuals in this age group go through the stage of forming habits and setting priorities, and they can be influenced by the surrounding society more than other groups [57,58,59]. Therefore, overcoming discouragement from the surrounding society can be complicated, which can result in acquiring misconceptions about the importance of physical activities.

The analysis of physical activity contexts indicates that males were more likely to exercise outdoors, with other people and via playing sports. In contrast, females preferred to exercise indoors, alone and via lifestyle activities. Many factors may contribute to these results, including the lack of suitable outdoor facilities for females, as reported by many researchers [18,60,61], and the fact that team sports are still not widely adopted among the female community in Saudi Arabia. On the other hand, football is the most prominent sport in Saudi Arabia, enjoyed by most Saudi males since childhood and frequently played outdoors despite the lack of appropriate outdoor facilities [25,62]. To the best of our knowledge, the differences mentioned above have not been well studied in previous literature and require further investigation and strategic development to promote physical activity opportunities in the Saudi population.

In terms of the contribution of physical activity context to health and well-being, many important findings emerged. The results show that the associations with general health are small, suggesting that the setting or mode of activity is less critical than the appropriate volume and intensity [5]. However, regarding the associations between physical activity contexts and well-being, the current study indicates that well-being was associated with exercising with others but not with exercising alone. Previous studies [29,63,64,65] have also demonstrated similar results. It is possible that social interaction, along with physical activity, may considerably contribute to these results, as many scholars have reported that social interaction has a beneficial influence on well-being [66,67,68].

Another important finding was that the relationship between exercising via sports and well-being is stronger than the relationship between lifestyle exercise and well-being; in addition, lifestyle exercise was not significant predictor of well-being. This result accords with other previous studies, which showed that the impact of exercising via playing sports on well-being is greater than that of lifestyle activities [69,70]. This finding may be explained by the fact that sports include the elements of fun and competition between people, which can contribute to enhancing well-being. The last finding from the analysis of the associations between physical activity contexts and well-being is that exercising outdoors was more strongly associated with well-being than exercising indoors. Similarly, indoor exercise was not a significant predictor of well-being. This is in accordance with a large body of research showing the positive impact of exercising in an outdoor environment on well-being [71,72,73]. All these findings confirm the importance of educating Saudis about the benefits of exercising with others in outdoor environments. Meanwhile, the Saudi government should be aiming to provide conducive environments for both males and females of different ages in all regions. One example of this is the redevelopment of the Wadi Hanifa valley in Riyadh, which involved the creation of a recreational area to enable physical activity for the whole community [74]. In neighbouring Qatar, the Umm Al Seneem park includes the world’s longest air-conditioned path designed for running and walking, along with fitness stations, play areas and a cycling track [75].

In relation to the impact of nature relatedness on health and well-being, the results of this study indicate that the associations between these variables were significant. Results from this study are similar to those reported in other studies, such as those conducted among Austrians, Australians and British citizens, which concluded that connection to nature can enhance well-being and health [33,76,77]. These results highlight that Saudis can benefit from being connected to nature; therefore, developing natural environments in all regions of Saudi Arabia should be a strategic target for the government.

Our study has several strengths. Notably, it is the first to investigate physical activity barriers in a representative sample of Saudi adults with specific attention on understanding whether different barriers were more relevant for females than for males. It is also the first study to examine the relevance of the outdoor environment and the connection with nature of residents to better understand how to improve approaches to the promotion of physical activity in Saudi Arabia since these have been identified as important for health and well-being promotion in some countries. To ensure a rigorous analysis, validated scales for assessing barriers, as well as physical activity, nature relatedness and well-being, were used. Existing validated Arabic versions of the IPAQ and WHO-5 were available to use, and a recognised process was followed to develop translations of the EBBS and NR-6 scales. Despite the strengths of this study, important limitations need to be acknowledged. While the sample was recruited by an international research agency to ensure national representativeness, it was based on the urban population only, and the results therefore may not represent rural residents. Similarly, although the sample was successfully balanced in terms of age and gender variables, it was skewed towards higher levels of education. Lastly, despite the large sample size, the number within some groups, such as older females, is small. Nonetheless, the data generated from this study provided valuable insights into current exercise contexts and barriers to help inform exercise promotion strategies.

## 5. Conclusions

Based on the study findings, it is notable that younger adults perceive greater obstacles to physical activity than middle-aged and older adults. This was observed for all categories of barrier and highlights the need to approach physical activity promotion differently for younger age groups. In addition, given the additional benefits of outdoor environments and connectedness with nature identified for well-being and general health, priority must be given by the Saudi government to adopt plans for developing outdoor environments and enhancing the efficiency of existing outdoor facilities.

## Figures and Tables

**Table 1:** Sociodemographic characteristics.

	Male (n = 640)	Female (n = 406)	Total (n = 1046)
Age group			
18–34 years	225	243	468
35–54 years	349	148	497
55+ years	66	15	81
Marital Status			
Single/divorced/widowed	172	189	361
Married	468	217	685
Educational Level			
Elementary/secondary/high school level	175	123	298
University level	442	273	715
Other (not specified)	23	10	33
Employment status			
Not working	162	275	437
Working part-time	91	65	156
Working full-time	387	66	453
Income level			
Less than USD 800	144	144	288
USD 800 to USD 3999	265	124	389
USD 4000+	128	52	180
Prefer not to say	67	37	104
Don’t know	36	49	85
Region			
East	40	14	54
West	254	178	432
Central	182	98	280
Other (North/South)	164	116	280

**Table 2:** Physical activity barriers by gender and age group.

	Exercise Milieu	Time Expenditure	Physical Exertion	Family Discouragement	Total Barriers	Weather Conditions
	Mean (SD)					
Total sample	2.29 ± 0.64	2.25 ± 0.73	2.24 ± 0.74	2.28 ± 0.81	31.79 ± 8.46	2.52 ± 0.91
Gender						
Male	2.26 ± 0.66	2.27 ± 0.74	2.20 ± 0.74 ^b^	2.25 ± 0.81	31.48 ± 8.75	2.51 ± 0.91
Female	2.33 ± 0.60	2.23 ± 0.72	2.31 ± 0.74 ^a^	2.34 ± 0.82	32.29 ± 7.98	2.54 ± 0.91
Age group						
18–34	2.39 ± 0.65 ^a^	2.33 ± 0.78 ^a^	2.37 ± 0.76 ^a^	2.37 ± 0.83 ^a^	33.19 ± 8.57 ^a^	2.62 ± 0.93 ^a^
35–54	2.22 ± 0.64 ^b^	2.20 ± 0.71 ^b^	2.16 ± 0.73 ^b^	2.23 ± 0.80 ^b^	30.84 ± 8.47 ^b^	2.45 ± 0.90 ^b^
55+	2.12 ± 0.50 ^b^	2.13 ± 0.56	2.07 ± 0.57 ^b^	2.10 ± 0.74 ^b^	29.52 ± 6.36 ^b^	2.41 ± 0.77
Gender and age group combinations						
18–34 years						
Male	2.40 ± 0.69	2.42 ± 0.80	2.38 ± 0.78	2.41 ± 0.83	33.65 ± 8.97	2.68 ± 0.94
Female	2.38 ± 0.61	2.25 ± 0.74	2.35 ± 0.74	2.34 ± 0.83	32.76 ± 8.17	2.56 ± 0.92
35–54 years						
Male	2.21 ± 0.66	2.20 ± 0.73	2.12 ± 0.73	2.20 ± 0.81	30.57 ± 8.81	2.43 ± 0.90
Female	2.25 ± 0.59	2.19 ± 0.67	2.26 ± 0.71	2.32 ± 0.79	31.48 ± 7.59	2.51 ± 0.90
55+ years						
Male	2.06 ± 0.46	2.11 ± 0.52	2.08 ± 0.50	1.96 ± 0.58 ^b^	28.83 ± 5.63	2.33 ± 0.71
Female	2.39 ± 0.57	2.24 ± 0.73	2.02 ± 0.85	2.73 ± 1.02 ^a^	32.53 ± 8.47	2.73 ± 0.96

Note: values in the same column with different superscripts are significantly different at *p* < 0.05; ^a^ superscript has the higher mean, ^b^ superscript has the lower mean.

**Table 3:** Physical activity context by gender and age group.

	Outdoors	Indoors	Alone	With Others	Part of Lifestyle	Organised Sport
	Mean ± SD					
Total sample	2.98 ± 0.76	2.71 ± 0.80	3.05 ± 0.73	2.60 ± 0.82	3.07 ± 0.70	2.75 ± 0.80
Gender						
Male	3.11 ± 0.69 ^a^	2.63 ± 0.80 ^b^	3.00 ± 0.72 ^b^	2.73 ± 0.80 ^a^	3.03 ± 0.73 ^b^	2.83 ± 0.77 ^a^
Female	2.79 ± 0.81 ^b^	2.84 ± 0.80 ^a^	3.13 ± 0.72 ^a^	2.41 ± 0.82 ^b^	3.13 ± 0.66 ^a^	2.63 ± 0.83 ^b^
Age group						
18–34 years	2.94 ± 0.80	2.86 ± 0.82 ^a^	3.16 ± 0.74 ^a^	2.62 ± 0.89	3.08 ± 0.73	2.81 ± 0.84
35–54 years	3.03 ± 0.72	2.62 ± 0.77 ^b^	2.97 ± 0.72 ^b^	2.61 ± 0.77	3.06 ± 0.69	2.72 ± 0.78
55+ years	2.97 ± 0.71	2.42 ± 0.71 ^b^	2.88 ± 0.58 ^b^	2.45 ± 0.66	3.06 ± 0.61	2.59 ± 0.71
Gender and age combinations						
18–34 years						
Male	3.17 ± 0.69 ^a^	2.79 ± 0.84	3.09 ± 0.75	2.86 ± 0.90 ^a^	3.05 ± 0.78	2.99 ± 0.79
Female	2.73 ± 0.83 ^b^	2.91 ± 0.80	3.23 ± 0.72	2.39 ± 0.83 ^b^	3.11 ± 0.68	2.65 ± 0.85
35–54 years						
Male	3.10 ± 0.69 ^a^	2.58 ± 0.77	2.97 ± 0.73	2.70 ± 0.74 ^a^	3.02 ± 0.71	2.77 ± 0.77
Female	2.86 ± 0.75 ^b^	2.73 ± 0.78	2.96 ± 0.69	2.40 ± 0.81 ^b^	3.15 ± 0.64	2.61 ± 0.80
55+ years						
Male	2.94 ± 0.64	2.38 ± 0.69	2.85 ± 0.54	2.37 ± 0.60	3.04 ± 0.63	2.60 ± 0.63
Female	3.08 ± 1.00	2.58 ± 0.79	3.00 ± 0.74	2.83 ± 0.84	3.17 ± 0.58	2.58 ± 1.00

Note: values in the same column with different superscripts are significantly different at *p* < 0.05; ^a^ superscript has the higher mean, ^b^ superscript has the lower mean.

**Table 4:** Correlations between well-being, nature relatedness and physical activity variables.

Variable		Mean	SD	1	2	3	4	5	6	7	8	9	10	11
1	Well-being	16.33	5.57											
2	General health	1.86	0.35	**0.32**										
3	Nature relatedness	21.16	4.71	**0.35**	**0.13**									
4	Physical activity #	1377	2274	**0.27**	**0.17**	**0.20**								
5	Exercise outdoors	2.98	0.76	**0.19**	0.04	**0.24**	**0.15**							
6	Exercise indoors	2.71	0.80	**0.11**	0.03	**0.12**	0.05	**−0.14**						
7	Exercise alone	3.05	0.73	0.06	0.02	**0.11**	**0.08**	**0.07**	**0.35**					
8	Exercise with others	2.60	0.82	**0.25**	**0.08**	**0.15**	**0.09**	**0.29**	**0.15**	**−0.18**				
9	Exercise as lifestyle	3.07	0.70	**0.12**	0.01	**0.15**	**0.09**	**0.10**	**0.21**	**0.25**	**0.18**			
10	Exercise via sports	2.75	0.80	**0.23**	**0.07**	**0.16**	**0.15**	**0.31**	**0.19**	0.04	**0.44**	**0.16**		
11	Total barriers	31.79	8.46	−0.01	**−0.09**	0.01	0.02	**0.08**	**0.31**	**0.17**	**0.21**	**0.14**	**0.18**	
12	Weather conditions	2.52	0.91	0	**−0.08**	0.05	0.01	**0.08**	**0.21**	**0.13**	**0.12**	**0.13**	**0.11**	**0.61**

Key: SD: standard deviation; # physical activity values are median and interquartile range. Bold highlights indicate significant correlations.

**Table 5:** Standardised regression coefficients for the relationship of predictor variables with psychological well-being.

Predictor Variable	Standardised Coefficient (β)	*p*-Value
Physical activity	0.17	0.001
Nature relatedness	0.25	<0.001
General health	0.23	<0.001
Exercise with others	0.13	0.004
Exercise outdoors	0.08	0.018
Exercise indoors	0.04	0.090
Exercise via sports	0.09	0.018
Exercise as lifestyle	0.03	0.322

## Data Availability

Not applicable.
